# Supplementary material for: Identification of photoperiod-induced specific miRNAs in the adrenal glands of Sunite sheep (Ovis aries)
Source: Front Vet Sci. 2022 Jul 22;9:888207. doi: 10.3389/fvets.2022.888207 (PMC9354845; doi:10.3389/fvets.2022.888207)
Supplement: Supplementary file 1 [file Data_Sheet_1.ZIP › Supplementary file/miRDeep2 file.zip!/miRDeep2 file/Novel_Structure/LP42A1/Novel_253.pdf]

Provisional ID	:	NC_019462.2_12782
Score total	:	1.6
Score for star read(s)	:	-1.3
Score for read counts	:	0
Score for mfe	:	1.3
Score for randfold	:	1.6
Score for cons. seed	:	
Total read count	:	113
Mature read count	:	113
Loop read count	:	0
Star read count	:	0

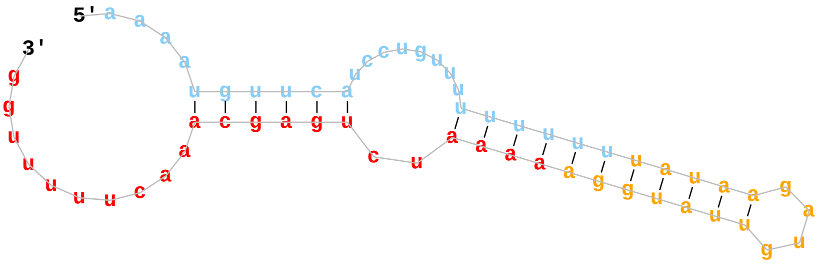

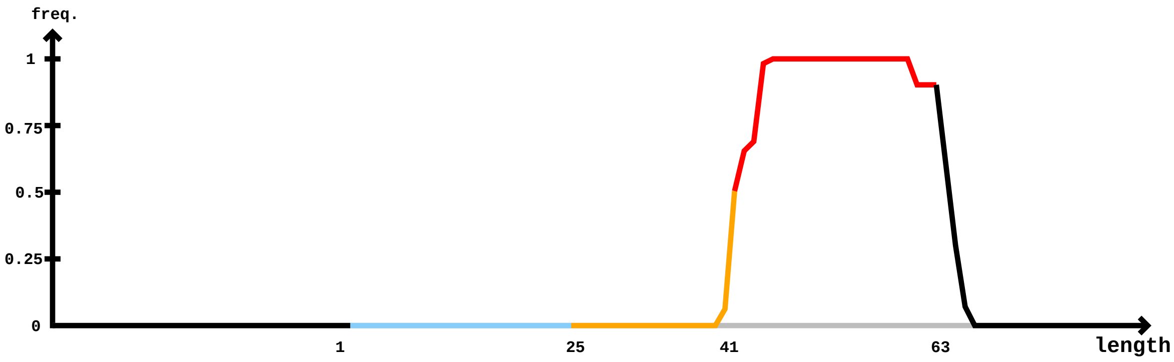

Star **Mature**

```
5'- uuuaaacaguaugauguauugguuuggccaaaauguucauccuguuuuuuuuuuauaagauguuauggaaaaaucugagcaaacuuuuuggccaauccaguauuucaguuca   -3'    exp
    ..........(((.(((((((.(((((((((.(((.....((((((((((((((((....)))))))))))...)))))))).))))))))).))))))).))).....   reads     mm        sample
    ....................................................................aaaaCcugagcaaacuuuu.........................   7         1         seq
    .....................................................................aaaaucugaAcaaacuuuu........................   4         1         seq
    .....................................................................aaaaucugaAcaaacuuuuugg.....................   45        1         seq
    .....................................................................aaaaucugaAcaaacuuuuuggc....................   1         1         seq
    ......................................................................aaaucugaAcaaacuuuuugg.....................   14        1         seq
    ......................................................................aaaucugagcaaacuuuuuggc....................   3         0         seq
    .......................................................................aaucugaAcaaacuuuuuggc....................   4         1         seq
    ........................................................................aucugaAcaaacuuuuuggcc...................   25        1         seq
    ........................................................................aucugaAcaaacuuuuuggcca..................   8         1         seq
    .........................................................................ucugaAcaaacuuuuuggc....................   1         1         seq
    .........................................................................ucugagcaaacuuuuuggcc...................   1         0         seq
```
